# Identification of the Regulators of Epidermis Development under Drought- and Salt-Stressed Conditions by Single-Cell RNA-Seq

**DOI:** 10.3390/ijms23052759

**Published:** 2022-03-02

**Authors:** Zhixin Liu, Chenxi Guo, Rui Wu, Jiajing Wang, Yaping Zhou, Xiaole Yu, Yixin Zhang, Zihao Zhao, Hao Liu, Susu Sun, Mengke Hu, Aizhi Qin, Yumeng Liu, Jincheng Yang, George Bawa, Xuwu Sun

**Affiliations:** 1 State Key Laboratory of Crop Stress Adaptation and Improvement, School of Life Sciences, Henan University, 85 Minglun Street, Kaifeng 475001, China; zxlsch2019@163.com (Z.L.); chenxi1445@163.com (C.G.); wuruiwr347538@sina.com (R.W.); wdj_3079@sina.com (J.W.); zhouyapinghenu@sina.com (Y.Z.); yxl86420@sina.com (X.Y.); zyx99292021@163.com (Y.Z.); zzh47091839@163.com (Z.Z.); lhao0520@126.com (H.L.); su17861523158@sina.com (S.S.); mengkehu218511@sina.com (M.H.); qin15939246835@sina.com (A.Q.); l13569882910@sina.com (Y.L.); 13458345215@sina.cn (J.Y.); ge.9410@yahoo.com (G.B.); 2 State Key Laboratory of Cotton Biology, School of Life Sciences, Henan University, 85 Minglun Street, Kaifeng 475001, China; 3 Key Laboratory of Plant Stress Biology, School of Life Sciences, Henan University, 85 Minglun Street, Kaifeng 475001, China

**Keywords:** PIFs, leaf, epidermal cell, development, drought, salt, scRNA-Seq

## Abstract

As sessile organisms, plants constantly face challenges from the external environment. In order to meet these challenges and survive, plants have evolved a set of sophisticated adaptation strategies, including changes in leaf morphology and epidermal cell development. These developmental patterns are regulated by both light and hormonal signaling pathways. However, our mechanistic understanding of the role of these signaling pathways in regulating plant response to environmental stress is still very limited. By applying single-cell RNA-Seq, we determined the expression pattern of *PHYTOCHROME INTERACTING FACTOR* (*PIF*) *1*, *PIF3*, *PIF4*, and *PIF5* genes in leaf epidermal pavement cells (PCs) and guard cells (GCs). PCs and GCs are very sensitive to environmental stress, and our previous research suggests that these PIFs may be involved in regulating the development of PCs, GCs, and leaf morphology under environmental stress. Growth analysis showed that *pif1/3/4/5* quadruple mutant maintained tolerance to drought and salt stress, and the length to width ratio of leaves and petiole length under normal growth conditions were similar to those of wild-type (WT) plants under drought and salt treatment. Analysis of the developmental patterns of PCs and GCs, and whole leaf morphology, further confirmed that these PIFs may be involved in mediating the development of epidermal cells under drought and salt stress, likely by regulating the expression of *MUTE* and *TOO MANY MOUTHS* (*TMM*) genes. These results provide new insights into the molecular mechanism of plant adaptation to adverse growth environments.

## 1. Introduction

The growth and development of plants are continually challenged by diverse environmental stressors, both biotic and abiotic [1,2,3,4,5]. Salinity, drought, and extreme temperature are the principal abiotic stress factors, which strongly affect the photosynthetic capacity, mineral utilization efficiency, pollen vitality, seed quality, and yield of higher plants [6,7,8,9,10]. In order to survive and successfully reproduce, plants have evolved sophisticated strategies for perceiving and appropriately adjusting to these environmental challenges [1,2,11,12]. Among these stress response mechanisms, two main strategies can be distinguished: stress resistance and stress tolerance. Resistance refers to plants’ mechanisms for preemptively avoiding a stressor. For example, cactus has made structural adjustments to its morphology, physiology, and metabolism to adapt to high temperature and aridity [13,14]. Tolerance refers to mechanisms that allow plants to grow and reproduce despite experiencing stress. For example, in response to drought stress, the majority of plants will reduce water loss by closing stomata so as to increase their survival probability.

Although morphological changes in response to environmental stress can occur due to passive selection, they can also occur through an active and adaptive process [12,15]. Stress leads to an imbalance in mitochondrial energy metabolism, a decline in photosynthetic efficiency, and an increase in cellular reactive oxygen species (ROS) [8,9,10,16,17]. These metabolic changes can further affect the physiological processes of plants, including obstructing the development of roots and leaves. Stress can also induce plants to produce stress-related hormones, such as abscisic acid (ABA), ethylene (ET), and gibberellins (GAs) [1,2,18,19,20]. These hormones are widely involved in regulating plant growth and development [21,22,23]. Therefore, under stress conditions, the production of these stress-related hormones can actively guide the growth and development of plants toward a state more conducive to survival.

Extensive studies have revealed the effects of drought and salt stress on the development of plant leaves [12,15,24,25,26]. There is a close relationship between the development of leaf epidermal cells and gross leaf morphology [27,28,29,30,31,32,33]. Meristemoid mother cells (MMCs) are the precursor to most differentiated leaf epidermal cells [34]. The differentiation of MMCs into daughter epidermal lineages is impacted by drought and salt stress through ROS production, brassinolide (BR), ET, and ABA [2]. In particular, the development and function of epidermal pavement cells (PCs) and guard cells (GCs) are very sensitive to external environmental conditions, and the differentiation and development of GCs are regulated by TMM and MUTE [34,35,36]. Many studies have revealed that auxin is involved in regulating the drought response of plants [37,38,39]. PIN1 is involved in regulating the development of leaf in response to auxin signaling [40,41]. The increased accumulation of auxin during drought stress not only supports plants’ tolerance to drought but also actives the auxin signaling, which may in turn rely on PIN1 to affect the development of leaf. Therefore, we speculate that drought and salt stress may affect both the development of leaf epidermal cells, including GCs and PCs and gross leaf morphology. However, our understanding of the molecular mechanism regulating the morphological development of plant leaves under stress is still very limited.

In our previous single-cell RNA sequencing (scRNA-Seq) study on cotyledon stomatal lineage cells, we found that *PHYTOCHROME INTERACTING FACTOR 4* (*PIF4*) and *PIF5* are highly expressed in MMCs and GCs and that they are key transcription factors that mediate the development of stomatal lineage cells [42]. Specifically, PIFs regulate protein PHYB, which plays a regulatory role in light signaling [43]. Light signaling plays a key role in regulating the growth and development of plant leaves [44,45], including stomatal lineage cells [46,47]. For example, compared with wild-type (WT) plants, the length to width ratio (L–W ratio) of both leaves and petioles in phyb mutants was increased, while in PHYB-overexpressing plants, the L–W ratio of leaves decreased and the petiole shortened [44,45]. Additionally, we previously found that PIF4 and WRKY33 can form a regulatory feedback loop mediating ROS homeostasis in plants under stress [48]. These previous results suggest that PIFs may regulate the development of leaf morphology and epidermal cells under drought and salt stress. Therefore, in this study, we systematically studied the effects of *pif1/3/4/5* on leaf epidermal cell development and leaf morphology under drought and salt stress conditions.

## 2. Results

### 2.1. Identification of the Cell Types of Cotyledons by scRNA-Seq

**Figure 1:**
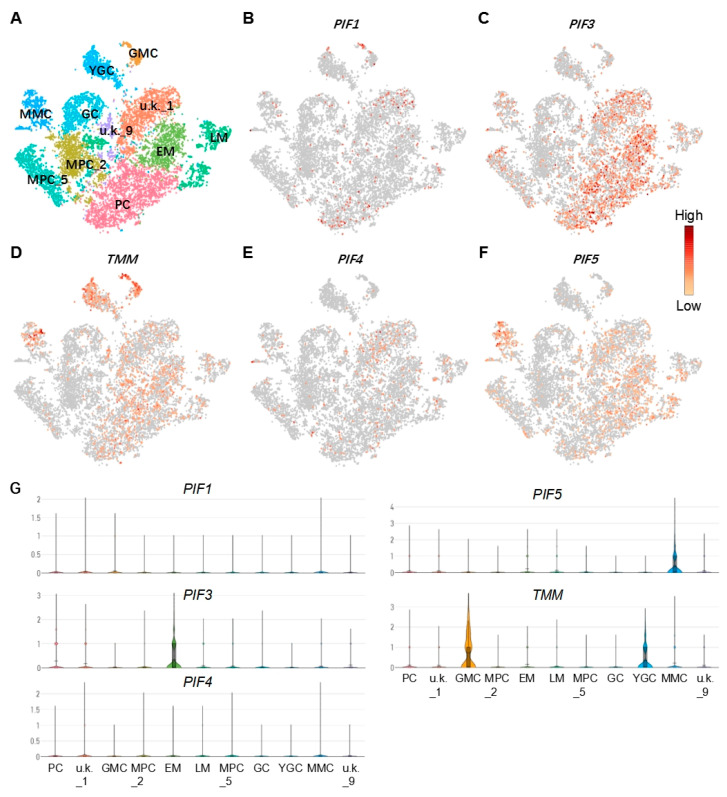
Analysis of the feature of *PIF* and *TMM* genes in epidermal cells. (**A**) A model demonstrates the distribution of cell types on tSNE plot; corresponding cell types are annotated on the plot. (**B**–**F**) Feature plots show the expression patterns of *TMM*, *PIF1*, *PIF3*, *PIF4*, and *PIF5*, respectively. (**G**) Violin plots show expression levels of *TMM*, *PIF1*, *PIF3*, *PIF4*, and *PIF5*, in each cell type. PC: pavement cell; M: meristemoid; GMC: guard mother cell; GC: guard cell; MMC: meristemoid mother cell; EM: early stage meristemoid; LM: late stage meristemoid; YGC: young guard cell; MPC: mesophyll cell; u.k.: unknown.

To investigate the potential regulator of the epidermal cells, we first determined the cell types based on previously produced scRNA-Seq data [42]. The cell types such as pavement cell (PC), guard mother cell (GMC), guard cell(GC), meristemoid mother cell (MMC), early stage meristemoid (EM), late stage meristemoid (LM), young guard cell (YGC), and mesophyll cell (MPC) were identified based on the known marker genes for the corresponding cell type. A cell cluster without a known marker gene was annotated as unknown (u.k.). Then, the differentially expressed genes (DEGs) in the corresponding cell type were screened as described in the Materials and Methods [42] (Table S1). From the DEGs, we found that PIF1, PIF3, PIF4, and PIF5 were expressed in both PCs and stomatal lineage cell populations, such as MMC, EM, and LM (Figure 1), suggesting that these PIFs may be involved in regulating the development of PC and GC.

### 2.2. Detection of the Specific Expression of PIF1/3/4/5 in Epidermal Cells by scRNA-Seq

**Figure 2:**
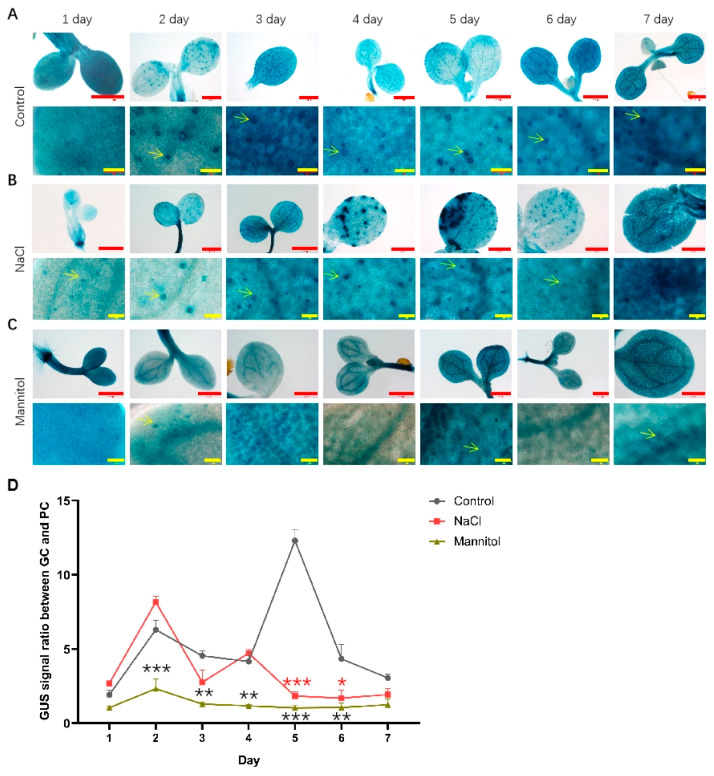
Analysis of the expression of PIF4pro:GUS after being treated with NaCl and mannitol. Analysis of the expression of PIF4pro:GUS in whole cotyledons (up panel) and lower epidermis during development of seedlings grown under control (**A**), NaCl (**B**), and mannitol (**C**) conditions, respectively. The seedlings of PIF4pro:GUS were grown in 1/2 MS plates plus 100 mM NaCl or 150 mM mannitol for 1–7 days, respectively; the seedlings of PIF4pro:GUS grown in normal 1/2 MS plates were used as controls. Then the seedlings were harvested and used for detecting the GUS activities by GUS staining, as described in Materials and Methods. Scale bar: red bar 1 mm, yellow bar 50 μm. (**D**) Statistical analysis of the ratio of GUS signal in GC vs. PC. The data were analyzed by one-way ANOVA following Brown–Forsythe test. *: *p* < 0.05; **: *p* < 0.01; ***: *p* < 0.001. Red star: NaCl vs. control; black star: mannitol vs. control.

In order to analyze the temporal and spatial expression dynamics of *PIF4* and *PIF5* under normal, drought, and salt treatment conditions, we used transgenic plants expressing PIF4pro:GUS and PIF5pro:GUS. As shown in Figure 2A, under normal growth conditions, there was no significant change in the expression level of PIF4pro:GUS in whole cotyledons from day 1 to day 7. By analyzing the expression of PIF4pro:GUS in the lower epidermis of cotyledons of seedlings, it was found that the GUS signal was significantly enhanced in GC from day 2. Under NaCl and mannitol treatment conditions, compared with that of control, the GUS signal of PIF4pro:GUS in whole cotyledons did not change significantly, but its relative ratio of GUS signals in GCs vs. PCs was decreased, suggesting that NaCl and mannitol treatment will inhibit the expression of PIF4pro: GUS in GC (Figure 2B,C).

**Figure 3:**
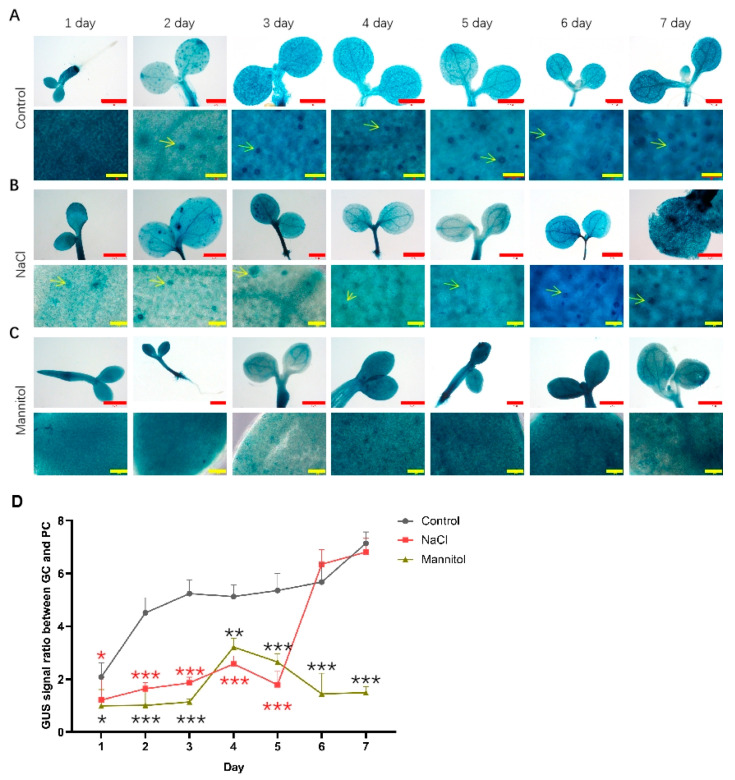
Analysis of the expression of PIF5pro:GUS after being treated with NaCl and mannitol. Analysis of the expression of PIF5pro:GUS in whole cotyledons (up panel) and lower epidermis during development of seedlings grown under control (**A**), NaCl (**B**), and mannitol (**C**) conditions, respectively. The seedlings of PIF5pro:GUS were grown in 1/2 MS plates plus 100 mM NaCl or 150 mM mannitol for 1–7 days, respectively; the seedlings of PIF5pro:GUS grown in normal 1/2 MS plates were used as controls. Then the seedlings were harvested and used for detecting the GUS activities by GUS staining as described in methods. Scale bar: red bar 1 mm, yellow bar 50 μm. (**D**) Statistical analysis of the ratio of GUS signal in GC vs. PC. The data were analyzed by one-way ANOVA following Brown–Forsythe test.*: *p* < 0.05; **: *p* < 0.01; ***: *p* < 0.001. Red star: NaCl vs. control; black star: mannitol vs. control.

As shown in Figure 3, the expression of PIF5pro:GUS in whole cotyledons did not change significantly from day 1 to day 7 with the progress of development. In the lower epidermis of cotyledons, the expression level of PIF5pro:GUS was also significantly enriched in GC. Similar to PIF4pro:GUS, compared with that of control, the expression level of PIF5pro:GUS did not change significantly under NaCl treatment conditions, but its expression in GC decreased significantly. Under mannitol treatment conditions, the expression level of PIF5pro:GUS in whole cotyledons was generally increased compared with the control, but its relative ratio of GUS signals in GCs vs. PCs was still decreased. These results show that NaCl and mannitol could specifically inhibit the expression of PIF4pro:GUS and PIF5pro:GUS in GC.

### 2.3. Drought and Salt Stress Affect the Development of Leaf Morphology

**Figure 4:**
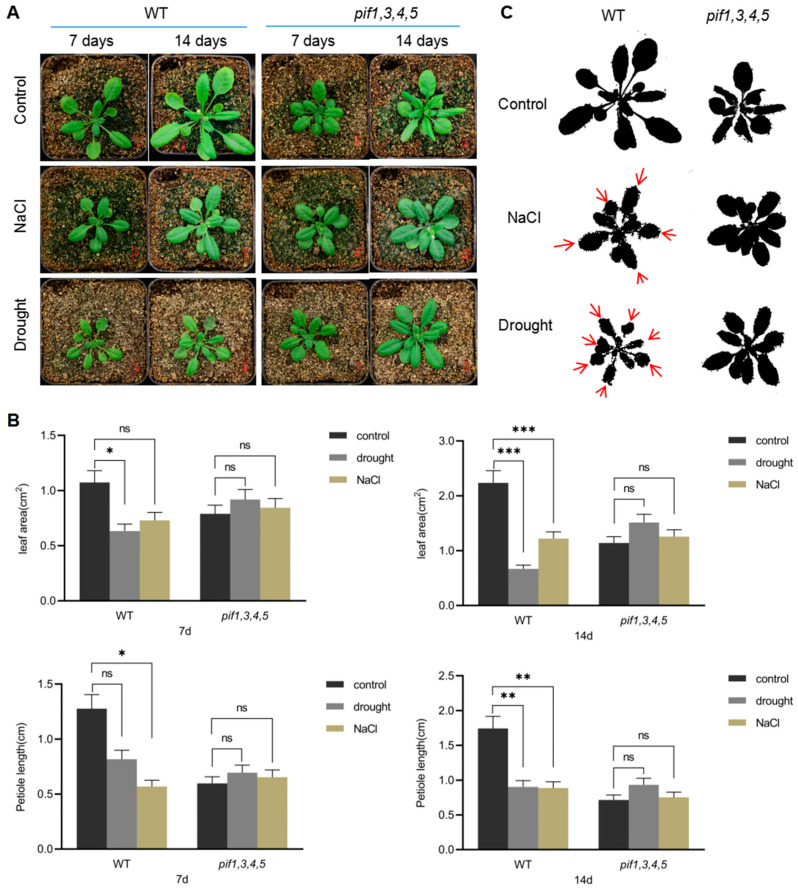

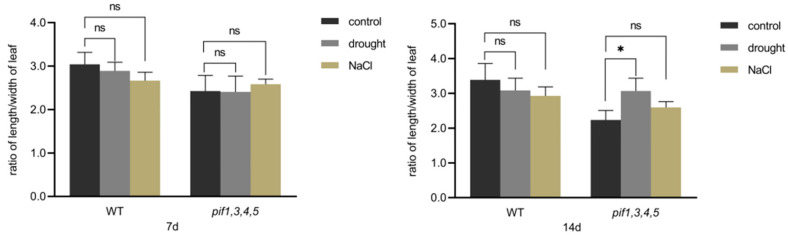
The changes in shape of leaves in response to NaCl and drought stresses. (**A**) The seedlings of *pif1/pif3/pif4/pif5* quadruple mutant (*pif1/3/4/5*) and WT were grown in soil for treatment with NaCl and drought for 7 days and 14 days, respectively; then the growth phenotype was recorded by photos; untreated seedlings were used as controls. Scale bar: 1 cm. (**B**) Statistical analysis of the leaf area, petiole length, and ratio of length/width of the leaf of WT and *pif1/3/4/5* quadruple mutant seedlings. The data were analyzed by one-way ANOVA following Brown–Forsythe test. ns: *p* > 0.05; *: *p* < 0.05; **: *p* < 0.01; ***: *p* < 0.001. (**C**) The diagrams demonstrate the changes in leaves of *pif1/3/4/5* and WT under control, NaCl, and drought conditions. Red arrows show the shrink direction of leaves of WT under NaCl and drought conditions. Scale bar: red bar 1 mm, yellow bar 50 μm.

In order to analyze the potential role of PIFs in regulating leaf development under drought and salt stress, we screened *pif1/pif3/pif4/pif5* quadruple mutant (*pif1/3/4/5*). There was functional redundancy among these PIFs [49], so we used *pif1/3/4/5* quadruple mutant in subsequent analyses. As shown in Figure 4A, compared with WT, *pif1/3/4/5* quadruple mutant plants under normal growth conditions had significantly shorter petioles and a significantly lower leaf L–W ratio. Between days 7 and 14 after treatment, WT leaf area increased 2 times, while *pif1/3/4/5* quadruple mutant leaf area increased only 1.6 times. Under NaCl and drought conditions, WT petiole length was significantly shortened, and the L–W ratio of leaves was also reduced, especially under NaCl treatment conditions (Figure 4A,B). Compared with WT, under NaCl and drought treatment conditions, the petiole length, leaf L–W ratio, and leaf area of *pif1/3/4/5* quadruple mutant plants showed little change (Figure 4A–C).

### 2.4. PIFs Are Involved in Regulating the Developmental Patterns of GC and PC

**Figure 5:**
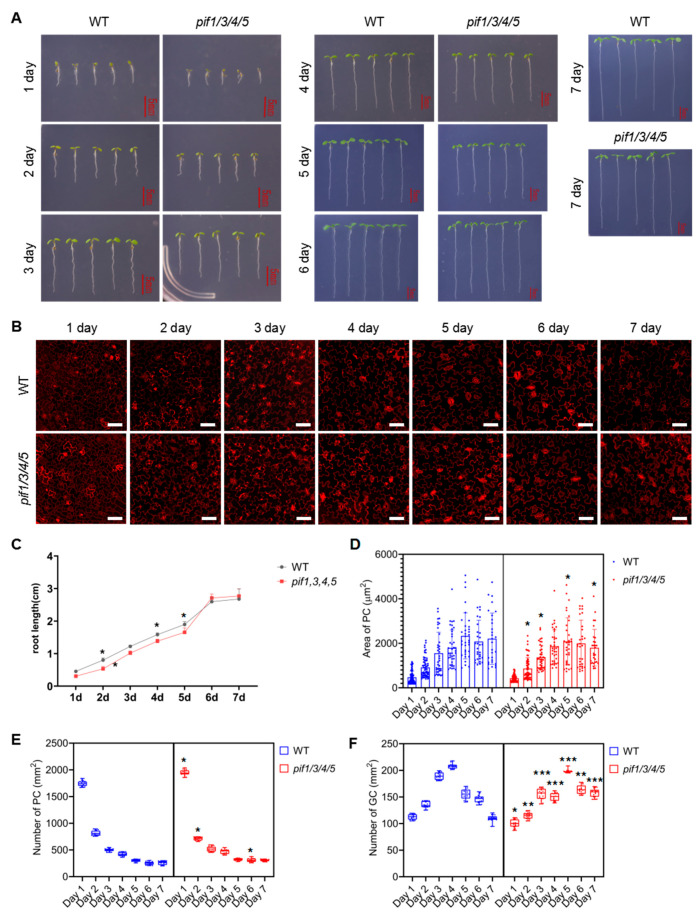
Analysis of the development of pavement cells (PCs) and guard cells (GCs) in *pif1/3/4/5* and WT under normal conditions. (**A**) Analysis of the growth of *pif1/3/4/5* and WT under control conditions. The seedlings of *pif1/3/4/5* and WT were grown in 1/2 MS plates for 1–7 days; the growth phenotype was recorded by photos; untreated seedlings were used as controls. Scale bar: 5 mm. (**B**) Detection of the development of PC and GC under the following conditions: The seedlings of *pif1/3/4/5* and WT were grown in 1/2 MS plates for 1–7 days and then harvested and stained with propidium (PI) for 30 min to stain the membranes. After PI staining, the lower epidermis of cotyledons of seedlings was subjected to detect PC and GC’s developmental state on a laser confocal microscope. Scale bar: 50 μm. (**C**) Statistical analysis of the root length of seedlings of *pif1/3/4/5* and WT (*n* = 5). (**D**) Statistical analysis of PC area in lower epidermis of cotyledons of seedlings of *pif1/3/4/5* and WT (*n* = 50). (**E**) Statistical analysis of the number of PC in lower epidermis of cotyledons of seedlings of *pif1/3/4/5* and WT (*n* = 10). (**F**) Statistical analysis of the number of GC in lower epidermis of cotyledons of seedlings of *pif1/3/4/5* and WT (*n* = 10). The data were analyzed by one-way ANOVA following Brown–Forsythe test. *: *p* < 0.05; **: *p* < 0.01; ***: *p* < 0.001.

In order to understand the role of PIFs in regulating GC and PC development, we analyzed the developmental dynamics of WT and *pif1/3/4/5* quadruple mutants during the early developmental stage of seedlings. As shown in Figure 5A,C, under normal growth conditions, the growth rate that was characterized by the roots length of *pif1/3/4/5* quadruple mutant seedlings was slightly slower than that of WT. Figure 5B shows the changing trend of PC and GC with elapsed time. Statistical analysis showed that in WT, the size of PCs quickly increased from day 1 to day 5, resulting in a rapid decrease in the number of PCs per unit area (Figure 5D,E). Compared with WT, the size of PCs in *pif1/3/4/5* quadruple mutant plants showed a similar growth trend, but individual cells were generally smaller than WT after day 5, resulting in a slightly higher number of PCs per unit area than WT (Figure 5D,E). The number of GCs per unit area in WT increased rapidly before day 4 and then decreased rapidly (Figure 5F). Compared with WT, *pif1/3/4/5* quadruple mutant plants had a greater number of GCs per unit area, and their increase was maintained through day 5 (Figure 5F).

### 2.5. Drought and Salt Stress Regulate the Developmental Patterns of GCs and PCs through PIFs

**Figure 6:**
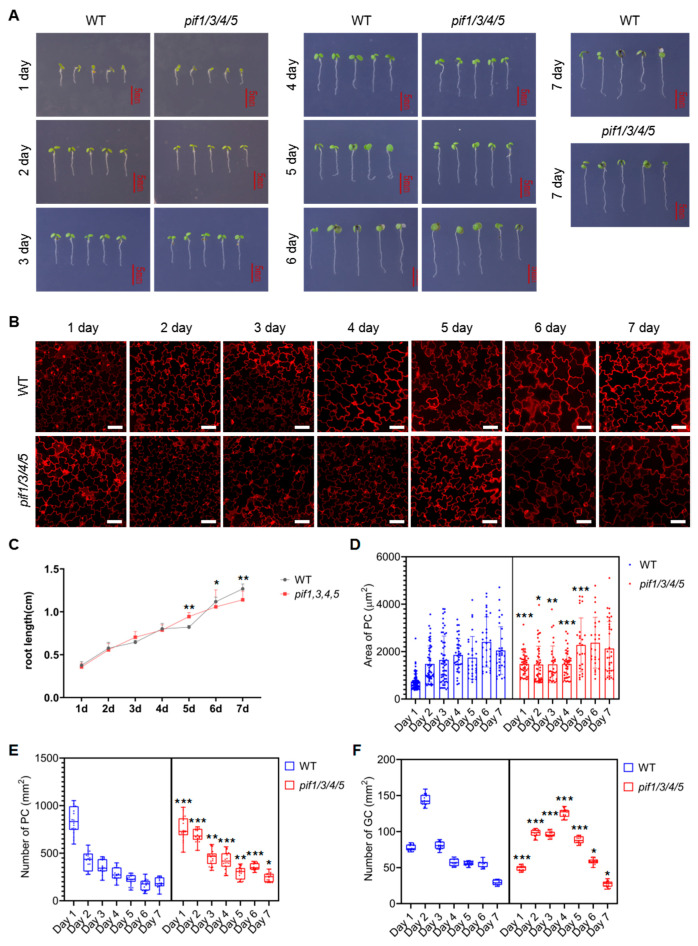
Analysis of the development of PC and GC in *pif1/3/4/5* and WT under NaCl condition. (**A**) Analysis of the growth of *pif1/3/4/5* and WT under control conditions. The seedlings of *pif1/3/4/5* and WT were grown in 1/2 MS plates plus 100 mM NaCl for 1–7 days. The growth phenotype was recorded by photos, and untreated seedlings were used as controls—scale bar: 5 mm. (**B**) Detection of the development of PC and GC under the following conditions. The seedlings of *pif1/3/4/5* and WT were grown in 1/2 MS plates plus 100 mM NaCl for 1–7 days and then harvested and stained with PI for 30 min to stain the membranes. After PI staining, the lower epidermis of cotyledons of seedlings was subjected to detect PC and GC’s developmental state on a laser confocal microscope. Scale bar: 50 μm. (**C**) Statistical analysis of the root length of seedlings of *pif1/3/4/5* and WT (*n* = 5). (**D**) Statistical analysis of PC area in lower epidermis of cotyledons of seedlings of *pif1/3/4/5* and WT (*n* = 50). (**E**) Statistical analysis of the number of PC in lower epidermis of cotyledons of seedlings of *pif1/3/4/5* and WT (*n* = 10). (**F**) Statistical analysis of the number of GC in lower epidermis of cotyledons of seedlings of *pif1/3/4/5* and WT (*n* = 10). The data were analyzed by one-way ANOVA following Brown–Forsythe test. *: *p* < 0.05; **: *p* < 0.01; ***: *p* < 0.001.

To analyze whether PIFs were also involved in regulating the differentiation and development of PCs and GCs under NaCl and drought conditions, we analyzed the growth, differentiation, and development dynamics of WT and *pif1/3/4/5* quadruple mutant seedlings. As shown in Figure 6A,C, the growth rate of *pif1/3/4/5* quadruple mutant seedlings was slightly faster than that of WT from day 1 to day 7 under NaCl treatment conditions. Figure 6B shows the developmental dynamics of PCs and GCs in WT and *pif1/3/4/5* quadruple mutant seedlings under NaCl treatment conditions. Statistical analysis showed that under NaCl treatment conditions, the size of PCs in WT plants increased gradually from day 1 to day 6 and stabilized on day 7 (Figure 6D). The number of PCs per unit area in WT plants under NaCl stress showed a rapid downward trend, which continued until day 7 (Figure 6E). Interestingly, we found that under NaCl treatment conditions, the size of PCs in *pif1/3/4/5* quadruple mutant seedlings was significantly larger than in WT on day 1, increased slowly from day 1 to day 4, increased rapidly from day 5 to day 6, and stabilized on day 7 (Figure 6D). In general, with the advance of growth time, the number of PCs per unit area in *pif1/3/4/5* quadruple mutant seedlings showed a gradual downward trend under NaCl treatment conditions and was generally higher than WT after day 2 (Figure 6E).

The analysis of the number of GCs per unit area indicated that under NaCl treatment conditions, the number of GCs per unit area in WT showed a rapid increase from day 1 to day 3, and then gradually decreased from day 3 until day 7 (Figure 6F). Compared with WT, the number of GC per unit area in *pif1/3/4/5* quadruple mutant seedlings showed an increase from day 1 to day 4, then decreased until day 7 (Figure 6F).

In addition to the change in PC and GC number and density, we also found that under NaCl treatment conditions, cellular morphology in both WT and *pif1/3/4/5* quadruple mutant was also affected. In PCs, lobal interdigitation and intercellular indentation became significantly atrophic (Figure 6B). In GCs, we observed more clusters of two or more GCs. Interestingly, under NaCl treatment conditions, there appeared to be defects in the development of stomata.

**Figure 7:**
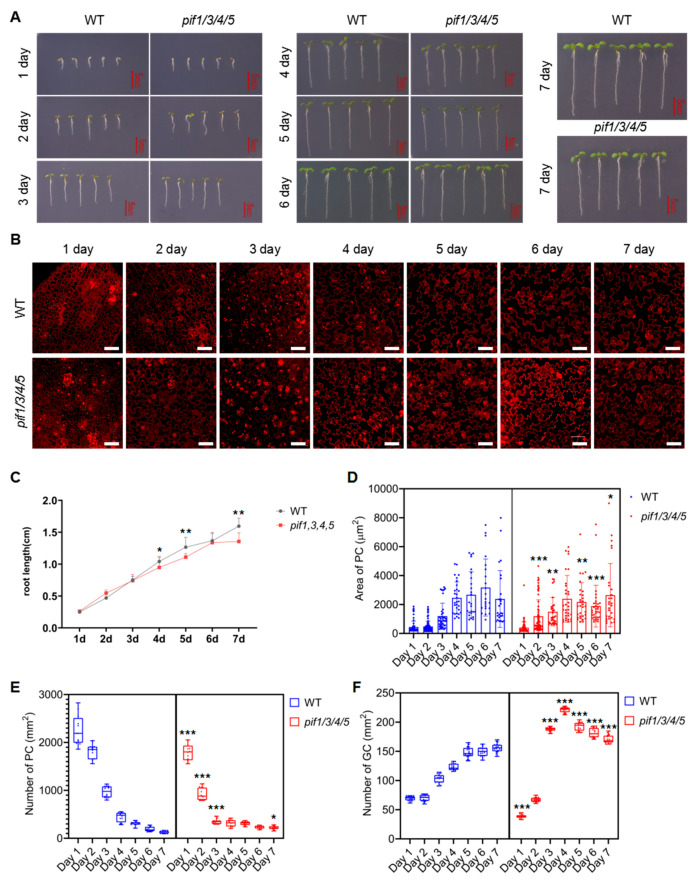
Analysis of the development of PC and GC in *pif1/3/4/5* and WT under mannitol conditions. (**A**) Analysis of the growth of *pif1/3/4/5* and WT under mannitol conditions. The seedlings of *pif1/3/4/5* and WT were grown in 1/2 MS plates plus 100 mM mannitol for 1–7 days. The growth phenotype was recorded by photos, and untreated seedlings were used as controls—scale bar: 5 mm. (**B**) Detection of the development of PC and GC under the following conditions: The seedlings of *pif1/3/4/5* and WT were grown in 1/2 MS plates plus 100 mM mannitol for 1–7 days and then harvested and stained with PI for 30 min to stain the membranes. After PI staining, the lower epidermis of cotyledons of seedlings was subjected to detecting the developmental state of PC and GC on laser confocal microscope. Scale bar: 50 μm. (**C**) Statistical analysis of the root length of seedlings of *pif1/3/4/5* and WT (*n* = 5). (**D**) Statistical analysis of PC area in lower epidermis of cotyledons of seedlings of *pif1/3/4/5* and WT (*n* = 50). (**E**) Statistical analysis of the number of PC in lower epidermis of cotyledons of seedlings of *pif1/3/4/5* and WT (*n* = 10). (**F**) Statistical analysis of the number of GC in lower epidermis of cotyledons of seedlings of *pif1/3/4/5* and WT (*n* = 10). The data were analyzed by one-way ANOVA following Brown–Forsythe test. *: *p* < 0.05; **: *p* < 0.01; ***: *p* < 0.001.

As shown in Figure 7A,C, compared with the control, the growth of WT and *pif1/3/4/5* quadruple mutant seedlings was significantly inhibited under mannitol treatment conditions. Under mannitol treatment conditions, the growth rate of *pif1/3/4/5* quadruple mutant seedlings was slightly faster than that of WT before day 3 (Figure 7A,C). After day 4, the growth rate in *pif1/3/4/5* quadruple mutant seedlings was slightly slower than that of WT (Figure 7A,C). The developmental dynamics of PCs and GCs in WT and *pif1/3/4/5* quadruple mutant seedlings under mannitol treatment conditions are shown in Figure 7B. Statistical analysis showed that under mannitol treatment conditions, the size of PCs in WT plants showed a gradual increase from day 1 to day 6 and stabilized on day 7 (Figure 7D). The number of PCs per unit area showed a rapid downward trend, which continued to day 7 (Figure 7E). Compared with WT, under mannitol treatment conditions, the size of PCs in *pif1/3/4/5* quadruple mutant seedlings showed a gradual increase from day 1 to day 4 and then remained stable (Figure 7D). The number of PCs per unit area showed an opposite trend with the area of PCs (Figure 7D,E). Overall, before day 4, the number of PCs in *pif1/3/4/5* quadruple mutant seedlings was significantly lower than that in WT, while after day 4, the number of PCs in *pif1/3/4/5* quadruple mutants was close to or slightly higher than that in WT (Figure 7D,E). The number of GCs per unit area in WT showed a gradual increase from day 1 to day 7 (Figure 7F). Compared with WT, under mannitol treatment conditions, the number of GCs per unit area in *pif1/3/4/5* quadruple mutant seedlings showed a rapid increase from day 1 to day 4 and a gradual decrease after day 4 (Figure 7E). Overall, after day 3, the number of GCs per unit area in *pif1/3/4/5* quadruple mutant seedlings was significantly higher than that in WT (Figure 7E). Mannitol treatment resulted in less significant changes to cellular morphology, and we still observed some cluster aggregation of GCs and defects in stomatal development in both WT and *pif1/3/4/5* quadruple mutant (Figure 7B).

### 2.6. PIFs Regulate the Developmental Patterns of GCs and PCs by Affecting the Expression of TMM and MUTE

**Figure 8:**
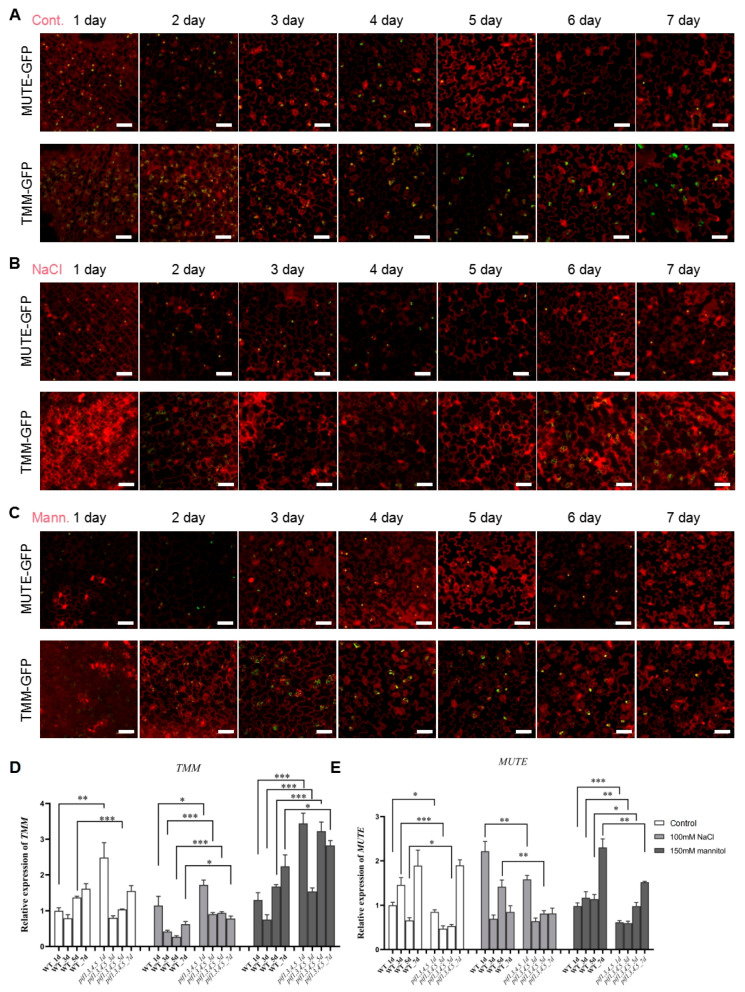
Analysis of the effects of NaCl and mannitol on the expression of *MUTE* and *TMM*. (**A**) The seedlings of MUTE-GFP and TMM-GFP were grown in 1/2 MS plates for 1–7 days, then were harvested and stained with PI for 30 min to stain the membranes. After PI staining, the lower epidermis of cotyledons of seedlings was subjected to detecting the expression of GFP signals on laser confocal microscope. Scale bar: 50 μm. (**B**) The seedlings of MUTE-GFP and TMM-GFP were grown in 1/2 MS plates plus 100 mM NaCl for 1–7 days, then were harvested and stained with PI for 30 min to stain the membranes. After PI staining, the lower epidermis of cotyledons of seedlings was subjected to detecting the expression of GFP signals on laser confocal microscope. Scale bar: 50 μm. (**C**) The seedlings of MUTE-GFP and TMM-GFP were grown in 1/2 MS plates plus 150 mM mannitol for 1–7 days and then harvested and stained with PI for 30 min to stain the membranes. After PI staining, the lower epidermis of cotyledons of seedlings was subjected to detecting the expression of GFP signals on laser confocal microscope. Scale bar: 50 μm. (**D**,**E**) qPCR analysis of the relative expression of *TMM* and *MUTE* under control, NaCl, and mannitol conditions, respectively. Relative expression indicates the mean value (±SD) of three independent experiments. The black stars represent student’s *t*-test of *pif1/3/4/5* vs. WT. *: *p* < 0.05; **: *p* < 0.01; ***: *p* < 0.001.

Considering the defects in stomatal development caused by NaCl and mannitol treatment, it may be that these treatments altered the expression of *TMM* and *MUTE*. In order to explore the mechanism by which NaCl and mannitol regulate the development of GC and PC, we analyzed the expression dynamic of *TMM* and *MUTE* under control, NaCl, and mannitol treatment conditions. As shown in Figure 8A, under control conditions, MUTE-GFP and TMM-GFP were specifically expressed in GCs, showing a decreasing and increasing trend with time. Under NaCl treatment conditions, MUTE-GFP showed a gradual downward trend from day 1 to day 7, while the expression of TMM-GFP showed a relatively stable trend (Figure 8B). Under mannitol treatment conditions, MUTE-GFP again showed a gradual downward trend from day 1 to day 7, while the expression of TMM-GFP showed a rapid jump and then a gradual downward trend (Figure 8C). From the results of GFP expression analysis, the expression of *TMM* was very sensitive to NaCl treatment. To examine whether PIF4 and PIF5 could regulate the expression of *TMM* and *MUTE*, qPCR analysis was performed. Compared with control, NaCl treatment resulted in significant down-regulation of *TMM* expression in WT but not in *pif1/3/4/5* quadruple mutant plants (Figure 8D). Meanwhile, mannitol treatment enhanced the expression of *TMM* in both WT and *pif1/3/4/5* quadruple mutant, compared with that of control (Figure 8D). Under mannitol conditions, the levels of *TMM* in *pif1/3/4/5* quadruple mutant were also higher than WT (Figure 8D). These results suggest that PIFs may negatively regulate the expression of *TMM*. For *MUTE*, its expression did not show significant changes under both NaCl and mannitol conditions (Figure 8E).

## 3. Discussion

### 3.1. Drought and Salt Stress Affect the Development of Leaf Morphology and Epidermal Cells

Drought and salt stress strongly inhibit plant growth [15,50,51]. In order to survive, plants will adapt to these environmental conditions by changing the growth and development pattern of leaves and roots [24,26,52]. In our work, we observed changes to leaf morphology and the shortening of petioles under salt and drought stress (Figure 4). Interestingly, further analysis found that drought and salt stress also had a strong impact on the developmental patterns of PCs and GCs (Figure 5, Figure 6 and Figure 7). Stomata serve as an important channel between plants and the external environment [34]. Under both salt and drought stress, stomatal development was significantly altered, with two or more stomata clustered together (Figure 6 and Figure 7), which was similar to the stomatal development phenotype of *tmm* mutant plants [34,35,36]. Especially under NaCl treatment conditions, we detected many developmentally defective stomata (Figure 6). The differentiation and development of PCs are closely related to GCs [45]. Therefore, when the development of GCs is affected, it will directly affect the differentiation and development of PCs. Previous studies have identified several key genes involved in regulating both gross leaf and GC development when plants are subjected to drought and salt stress [12,15,24,25,26,50,52,53,54,55]. For instance, NbPHAN, a MYB transcriptional factor, has been found to be involved in regulating leaf development under water deficit. NbPHAN-silenced plants show severe wilting and increased rate of water loss when grown under drought stress [52]. Another example is drought sensitive 8 (DS8) [54], a Nck-associated protein 1 (NAP1)-like protein and a component of the SCAR/WAVE complex, which was found to be required for regulation of leaf cuticle development and stomatal density and closure activity. *Ds8* antisense mutants exhibit increased drought sensitivity, along with impaired stomatal closure and elevated ABA levels [54]. These studies show that plants actively regulate their development patterns as a key strategy for dealing with environmental stress.

### 3.2. Light Signaling Is Involved in Regulating Leaf Development under Drought and Salt Stress Conditions

Light signaling plays a key role in regulating plant growth and development [44]. However, it is unknown whether light signaling is involved in regulating plant growth and development patterns under salt and drought stress. Many studies have shown a close interaction between light signaling and hormone signaling pathways in plants [56,57,58,59,60,61,62]. For example, light signaling affects the growth and development of plants through the interaction of ABA, BR, ET, and auxin (AUX) signals [60]. Light signaling also plays an important role in regulating the development of GCs and PCs in plants [35,46,47]. ABA, BR, ET, and AUX signaling are also widely involved in regulating the development of GCs and PCs [34]. It is well known that under drought and salt stress, plants can quickly induce the production of ABA, BR, ET, and AUX, thus activating the signaling mediated by these hormones. Once these signaling pathways are activated, they will interact with light signaling to coordinate the regulation of plant growth and development patterns. Early studies have found that PIN1-mediated AUX signaling is involved in the regulation of PC development patterns and shape [63]. Under salt treatment conditions, we observed changes in PC development pattern and shape (Figure 6), suggesting that salt stress may regulate PC development through AUX-mediated signaling.

### 3.3. PIF4 and PIF5 Are Involved in Regulating the PC and GC Development by Mediating the Expression of MUTE and TMM under Drought and Salt Stress Conditions

In this study, we identified a list of genes highly expressed in PC by scRNA-seq data analysis. Among them, PIF4 and PIF5 were selected to investigate their potential roles in the regulation of PC and GC development. Growth analysis indicated that the pif1/3/4/5 quadruple mutant showed tolerance to drought and NaCl stress, compared with WT. Further analysis found that the development of GC and PC was sensitive to NaCl and drought stresses (Figure 5, Figure 6 and Figure 7). Because MUTE and TMM are important regulators of GC development [34,35,36], we therefore detected the expression of MUTE and TMM under drought and NaCl stress conditions. The qPCR analysis indicated that the expression level of TMM was changed under NaCl and mannitol conditions (Figure 8D). Compared with that of WT, the levels of TMM expression in *pif1/3/4/5* quadruple mutant was relatively higher, indicating that PIF4 and PIF5 maybe involved in negative regulation of TMM expression in response to NaCl and drought stress. For MUTE, consistent with the GFP signals, its expression showed relative stability under NaCl and mannitol conditions (Figure 8E). These results reveal the new roles of PIF4 and PIF5 in the regulation of PC and GC development by mediating the expression of TMM.

## 4. Materials and Methods

### 4.1. Plant Materials and Growth Conditions

Wild-type (WT) Arabidopsis (Arabidopsis thaliana) Columbia ecotype (Col-0) was used in this study. The *pif1*, *pif3*, *pif4*, *pif5* quadruple mutant ((*pif1-1*; *pif3-7*; *pif4-2*; *pif5-3* (CS66047)), PIF4pro:GUS (N69168), and PIF5pro:GUS (N69170) were obtained from the Arabidopsis Biological Resource Center (ABRC). All mutants and WT Arabidopsis were grown in an artificial climate chamber under the growth conditions of 21–23 °C, 100 μmol photons m-2s-1 (normal light treatment), 16 h light/8 h dark, 60–70% humidity. For NaCl and mannitol treatments, the seedlings were grown on 1/2 MS medium plates containing 100 mM NaCl or 150 mM mannitol for 1–7 days.

### 4.2. GUS Staining and Histological Analysis

Histochemical GUS staining was performed as described previously [64]. Samples were fixed in 90% acetone at −20 °C, rinsed four times with 0.1 M sodium phosphate buffer (pH 7.4), and then incubated in X-Gluc solution (0.1 M sodium phosphate (pH 7.4), 3 mM potassium ferricyanide, 0.5 mM potassium ferrocyanide, 0.5 g L^−1^ 5-bromo-4-chloro-3-indolyl-β-d-glucuronide cyclohexilammonium salt) at 37 °C. After staining, samples were incubated in methanol to remove chlorophyll and then mounted in the clearing solution (a mixture of chloral hydrate, water, and glycerol in a ratio of 8:2:1). Observation was performed using a stereomicroscope (MZ16F, Leica Microsystems, Germany) or a microscope equipped with Nomarski optics (BX51, Olympus Co., Ltd., Tokyo, Japan). The signal intensity of GUS was analyzed with ImageJ program. The relative ratio of GUS signals in GC vs. PC was analyzed based on the results of ImageJ.

### 4.3. Microscopy

The seedlings were stained with 10 g/mL propidium (PI) (Sigma, Darmstadt, Germany) for 1 min before imaging. For confocal microscopy, fluorescence in roots was detected using a confocal laser scanning microscope (LSM980, Zeiss, Jena, Germany). PI signal was visualized using wavelengths of 610 to 630 nm. GFP was observed using wavelengths of 510 to 530 nm. Images and GFP intensities were processed using Zeiss Confocal Software.

### 4.4. Total RNA Extraction and qPCR Analysis

Total RNA was extracted with FastPure Plant Total RNA Extraction kit (Cat. No. DC104, Vazyme; Nanjing, China). Total RNA was treated with DNaseI (Vazyme; Nanjing, China) for 30 min to remove the remaining DNA; then, the cDNA was synthesized with HiScript II One-Step RT-PCR Kit (Cat. No. P611, Vazyme; Nanjing, China); qRT-PCR was performed with the corresponding primers (Supplementals Table S1). qPCR run was performed on a CFX 96 (Bio-Rad, Herculesm, CA, USA) with the following cycle parameter: 95 °C for 30 s, 35 cycles of 95 °C for 30 s, 55–56 °C for 15 s, and 72 °C for 15 s.

### 4.5. Identification of the Genes Highly Expressed in Corresponding Cell Type

The average expression and dispersion were briefly calculated for all genes, which were subsequently placed into 10 bins based on expression. Principal component analysis (PCA) was performed to reduce the dimensionality on the log-transformed gene-barcode matrices of the most variable genes. Cells were clustered via a graph-based approach and visualized in two dimensions using tSNE. A likelihood ratio test, which simultaneously tests for changes in mean expression and percentage of cells expressing a gene, was used to identify significantly differentially expressed genes (DEGs) between clusters. We used the FindAllMarkers function (test.use = bimod, logfc.thresold = 0, min.pct = 0.25) in Seurat to identify DEGs of each cluster. For a given cluster, FindAllMarkers identified positive markers compared with all other cells.

## Data Availability

Not applicable.

## Supplementary Materials

The following supporting information can be downloaded at: https://www.mdpi.com/article/10.3390/ijms23052759/s1.
